# Characterization of Frond and Flower Development and Identification of FT and FD Genes From Duckweed *Lemna aequinoctialis* Nd

**DOI:** 10.3389/fpls.2021.697206

**Published:** 2021-10-11

**Authors:** Akiko Yoshida, Ken-ichiro Taoka, Aoi Hosaka, Keisuke Tanaka, Hisato Kobayashi, Tomoaki Muranaka, Kiminori Toyooka, Tokitaka Oyama, Hiroyuki Tsuji

**Affiliations:** ^1^Kihara Institute for Biological Research, Yokohama City University, Yokohama, Japan; ^2^NODAI Genome Research Center, Tokyo University of Agriculture, Tokyo, Japan; ^3^Department of Embryology, Nara Medical University, Nara, Japan; ^4^Faculty of Agriculture, Kagoshima University, Kagoshima, Japan; ^5^Technology Platform Division, Mass Spectrometry and Microscopy Unit, RIKEN Center for Sustainable Resource Science, Yokohama, Japan; ^6^Department of Botany, Graduate School of Science, Kyoto University, Kyoto, Japan

**Keywords:** duckweed, flowering, FT, transcriptome, photoperiod, *Lemna aequinoctialis*, FD

## Abstract

Duckweeds (*Araceae: Lemnoideae*) are aquatic monocotyledonous plants that are characterized by their small size, rapid growth, and wide distribution. Developmental processes regulating the formation of their small leaf-like structures, called fronds, and tiny flowers are not well characterized. In many plant species, flowering is promoted by the florigen activation complex, whose major components are florigen FLOWERING LOCUS T (FT) protein and transcription factor FD protein. How this complex is regulated at the molecular level during duckweed flowering is also not well understood. In this study, we characterized the course of developmental changes during frond development and flower formation in *Lemna aequinoctialis* Nd, a short-day plant. Detailed observations of frond and flower development revealed that cell proliferation in the early stages of frond development is active as can be seen in the separate regions corresponding to two budding pouches in the proximal region of the mother frond. *L. aequinoctialis* produces two stamens of different lengths with the longer stamen growing more rapidly. Using high-throughput RNA sequencing (RNA-seq) and *de novo* assembly of transcripts from plants induced to flower, we identified the *L. aequinoctialis FT* and *FD* genes, whose products in other angiosperms form a transcriptional complex to promote flowering. We characterized the protein-protein interaction of duckweed FT and FD in yeast and examined the functions of the two gene products by overexpression in *Arabidopsis*. We found that *L. aequinoctialis* FTL1 promotes flowering, whereas FTL2 suppresses flowering.

## Introduction

Duckweeds (*Araceae: Lemnoideae*) are small, rapidly growing aquatic monocot plants that can be vegetatively propagated in axenic culture (Ziegler et al., 2015). These characteristics have attracted special attention in the fields of plant genomics, biotechnology, physiology, and developmental biology (Appenroth et al., 2015). The genome size of members of the *Lemnoideae* ranges from 150 Mb in *Spirodela polyrhiza* to 1.9 Gb in *Wolffia arrhiza* (Kim et al., 2010; Wang et al., 2011, 2014; Van Hoeck et al., 2015; Ernst, 2016; Michael et al., 2017, 2021). In *S. polyrhiza* populations, low genetic variation is associated with a low mutation rate in this species (Xu et al., 2019). In the field of biotechnology, duckweeds are recognized as ideal candidates for producing proteins and chemical components for human consumption because of the fast population doubling time and wide distribution of the plant worldwide (Appenroth et al., 2015). Duckweeds are also attractive model plants for physiological research, such as for examining circadian clock regulation by light at the cellular level (Muranaka and Oyama, 2016). In contrast, duckweed development is not well characterized despite its attention from an evolutionary developmental view (Lemon and Posluszny, 2000). Duckweed shoots develop a small organ called a frond, whose nature is still in debate as to whether it is a leaf homolog or a combined leaf and stem. Duckweeds develop tiny flowers in the axil of the frond; however, flower development and the regulation of flowering at the molecular level have not been intensely investigated.

Studies of model plant species have revealed the molecular basis of flowering regulation (Tsuji et al., 2013). Under an inductive photoperiod, the expression of genes encoding FLOWERING LOCUS T (FT), a systemic flowering signal in plants, is activated in leaves (Kardailsky et al., 1999; Kobayashi et al., 1999; Abe et al., 2005; Wigge et al., 2005). FT protein is transported from the leaves to the nuclei of shoot apical meristem (SAM) cells, where FT forms florigen activation complexes (FAC) composed of 14-3-3 protein and FD, a basic leucine-zipper (bZIP) domain-containing transcription factor (Taoka et al., 2011; Collani et al., 2019). The FAC activate downstream genes including the MADS-box transcription factors *APETALA1* (*AP1*)/*FRUITFUL* (*FUL*) and *SUPPRESSOR OF OVEREXPRESSION OF CONSTANS1* (*SOC1*) that strongly promote the transition of the shoot apex from the vegetative stage to the reproductive stage for floral organ formation. This regulatory process required for flowering is conserved across diverse plant species including tomato, poplar, and maize (Park et al., 2014; Tylewicz et al., 2015; Sun et al., 2020). In duckweeds, however, the expression and function of these flowering genes are not well characterized.

In this study, we characterized the course of developmental changes during frond development and flower formation in *Lemna aequinoctialis* Nd. In *L. aequinoctialis*, flowering is induced by short days (Yukawa and Takimoto, 1976). Thus, this species is suitable for our developmental characterization. Using high throughput RNA sequencing (RNA-seq) of plants induced to flower by short days, we identified duckweed orthologs of *FT* and *FD* genes. We further characterized the interaction of the orthologous gene products to form FAC and function in heterologous systems. Our results suggest that *L. aequinoctialis* FTL1 promotes flowering, whereas FTL2 suppresses flowering.

## Materials and Methods

### Plant Materials and Growth Conditions

*Lemna aequinoctialis* Nd was maintained in NF medium (Muranaka et al., 2015). Colonies were grown in 40 ml of NF medium in 90 x 20 mm Petri dishes under short-day (8-h light/ 16-h dark cycles) or long-day (16-h light/8-h dark cycles) conditions. The growth temperature was maintained at 22 ± 1°C. *Arabidopsis* Col-0 (control) and transgenic *Arabidopsis* plants were grown in long-day conditions (16-h light/8-h dark cycles) at 23°C.

### Morphological Analysis

Fronds were photographed using a stereomicroscope (Olympus SZ61, Japan). For the scanning electron microscope (SEM) observations, fronds were fixed in 2.5% glutaraldehyde overnight at 4°C and dehydrated in a series of ethanol solutions. The final ethanol solution was substituted with 3-methyl butyl acetate, after which the samples were dried at their critical point, sputter-coated with platinum, and observed with a SEM (model Hitachi SU-1510, RIKEN) at an accelerating voltage of 5 kV.

### Ethynyl Deoxyuridine Staining

*Lemna aequinoctialis* Nd was cultured overnight in 10 ml of NF medium that included 10 mM ethynyl deoxyuridine (EdU). EdU-labeled fronds were then washed and observed by using an imaging kit (Click-iTTM EdU Alexa FluorTM 488), according to the instructions of the manufacturer.

### RNA Extraction and RNA-seq Analysis

We collected triplicate samples on day 0 and 5, 10, and 13 days after initiating the short-day treatment. Total RNA from *L. aequinoctialis* was isolated using an RNeasy Plant Mini Kit (Qiagen), and cDNA was synthesized with a SuperScript™ First-Strand Synthesis System for RT-PCR (Thermo Fisher Scientific) according to the instructions of the manufacturer. TruSeq RNA libraries were prepared according to the protocol of the manufacturer (TruSeq RNA Library Prep Kit v2, Illumina, San Diego, CA, USA). Libraries with an average insert size of 156 budding pouch (bp) were sequenced on the NextSeq500 System (Illumina) according to the instructions of the manufacturer. The read data were deposited to DDBJ (DRA Accession DRA011840).

Trimmomatic 0.39 software was used with the following options: “ILLUMINACLIP:TruSeq3-PE.fa:2:30:10 HEADCROP:10 LEADING:20 TRAILING:20 SLIDINGWINDOW:4:15 MINLEN:36” (Bolger et al., 2014). Reads that contain Poly-A or Poly-T sequences with more than 25nt were removed using seqkit. To exclude the reads derived from tRNA, rRNA, and chloroplasts, the reads were mapped to tRNA (Cognat et al., 2013), Embryophytic rRNA (Quast et al., 2013; Yilmaz et al., 2014), and chloroplast genome of *Lemna minor* (Mardanov et al., 2008) using HISAT2 (Kim et al., 2019). The Read 1 and Read 2 sequences were separately aligned by treating as single-end mode. The unmapped reads were extracted from the BAM files using SAMTools (Li et al., 2009) with the following options: “view -b -f 4,” then converted to fastq files with bamToFastq command of bedtools (2.28.0) (Quinlan and Hall, 2010). The unpaired reads from the extracted reads were excluded with seqkit (0.16.1) (Shen et al., 2016). The remaining reads were used for downstream analyses. A *de novo* transcriptome was assembled with Trinity (v2.8.5) using the default parameters (Grabherr et al., 2011).

### Gene Annotation

Open reading frames (ORFs) in all assembled contigs were extracted using TransDecoder.LongOrfs script with default parameters, which defines sequences as ORFs when lengths are equivalent to at least 100 amino acids (TransDecoder Release v5.5.0). Orthologous proteins of the defined ORFs were searched against the Swiss-Prot database (2021_3) by BLASTp with a 10^−5^ e-value cutoff (Altschul et al., 1990). Proteins with the lowest e-values were defined as orthologous proteins of the defined ORF. Protein domains in the defined ORFs were also searched using HMMER (Eddy, 2009) software against Pfam-A.hmm (2021_3) with default parameters. Protein-coding regions were predicted using TransDecoder.Predict script based on the results of BLASTp and HMMER searches. Gene ontology (GO) terms for each gene were determined based on the GO terms of the Swiss-Prot annotation.

### Detection of Differentially Expressed Genes

The align_and_estimate_abundance.pl script from the Trinity package (v.2.4.0) was applied to align reads to the *de novo* transcriptome with Bowtie (version 1.1.2) (Langmead, 2010) and to estimate the transcript abundance with RSEM (version 1.3.0) (Li and Dewey, 2011). An ANOVA-like test was used to detect differentially expressed genes (DEGs) at any time point after beginning the short-day treatment with edgeR (3.28.1) (Robinson et al., 2010).

To group DEGs with similar expression patterns, K-Means clustering (K = 6) was applied using Complexheatmap (2.2.0) (Gu et al., 2016).

For genes within each group, we used topGO (2.38.1) to find statistically overrepresented GO terms of biological processes compared to all annotated genes by a Fisher's exact test (*P* < 0.05).

### Multiple Sequence Alignment and Phylogenetic Analysis

Protein sequences of FD from *L. aequinoctialis* and other plant species were aligned to create a phylogenetic tree using MUSCLE (Edgar, 2004), to reconstruct the phylogeny only based on conserved residues in the alignment. We trimmed the original alignment using the program trimAl (version 1.2, Capella-Gutiérrez et al., 2009) and then the phylogeny was reconstructed based on the trimmed alignment. A phylogenetic analysis was conducted using the MEGA X program with 500 bootstrap replications. A maximum-likelihood tree was constructed using the Jones-Taylor-Thornton (Jones et al., 1992) model with the same alignment file. For protein sequence alignment of FT and FD to identify conserved motifs, the CLUSTAL W program was used.

### cDNA Cloning

The coding regions of duckweed FT and FD-like genes were PCR-amplified from *L. aequinoctialis* cDNA with PrimeSTAR GXL polymerase (TaKaRa) according to the instructions of the manufacturer. The amplified DNAs were cloned into the entry vector, pENTR-D-TOPO (Thermo Fisher SCIENTIFIC) using NEBuilder HiFi DNA Assembly Master Mix (New England Biolabs). The nucleotide sequences of the constructs were confirmed by sequencing. Primers for PCR amplification were designed according to data from *de novo* RNA-seq analyses. For LaFDL1, the following primers were used to amplify the coding sequences for cloning.

AoFD1-F: 5'-TCCGCGGCCGCCCCCTTCACCATGCGGCACCATCAGAAGCAAC-3'

pEN-AoFD-R2:

5'- TGGGTCGGCGCGCCCACCCTTTTGCACTCAAAAGGGTGCGG-3'

### Plasmid Construction

A Gateway-compatible destination vector, pGWB602 (Nakamura et al., 2010), was used to construct transgenic *Arabidopsis* plants. For the yeast two-hybrid assays, Gateway-compatible destination vectors, pBTM-GW and pVP16-GW (Taoka et al., 2011), were used. FT and FD coding regions were transferred from pENTR-D-TOPO to these destination vectors using the Gateway LR Clonase II enzyme mix (Thermo Fisher Scientific), according to the instructions of the manufacturer.

### Generation of Transgenic Arabidopsis and Flowering Time Analysis

*Agrobacterium* EHA105 was transformed with pGWB602 plasmids containing the *LaFTL* coding regions. *Arabidopsis* Col-0 plants were transformed by the floral dip method as described by Clough and Bent (1998). To select transgenic plants, the transformed seeds were germinated and grown on 1/2 x MS medium containing 10 mg/L glufosinate for 5 days. The surviving seedlings were transferred to soil and grown under long days (LD) conditions. To eliminate any escaped plants, the soil-grown plants were sprayed with 0.1% (w/v) BASTA (BASF) every 2 days for a week. For flowering time analysis, more than 10 independent transgenic lines (from 12 to 41 lines) from the T1 generation were used to count the number of rosette leaves at bolting.

### Yeast Two-Hybrid Assay

The LexA-based assay was performed essentially as described in a previous study (Taoka et al., 2011). Yeast transformation was accomplished using a Frozen-EZ Yeast Transformation II Kit (Zymo Research), according to the instructions of the manufacturer. Transformed yeast colonies were selected on synthetic complete medium without uracil, tryptophan, and leucine (SC-UWL) and grown on SC-UWL medium without histidine (SC-UWLH) and containing 10 mM of 3-amino-1,2,4-triazole (3-AT) for growth analysis.

## Results

### *Lemna aequinoctialis* Frond Development

**Figure 1:**
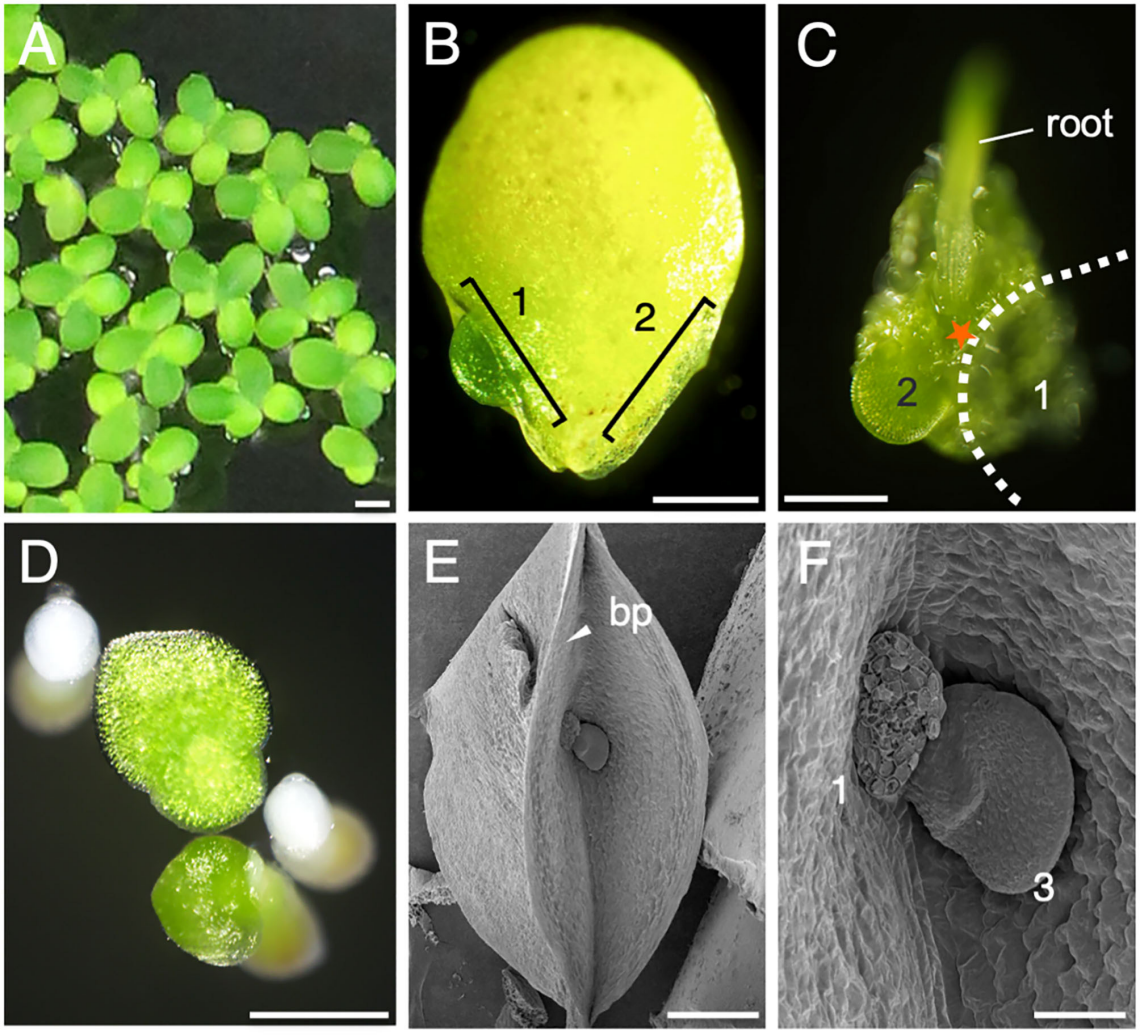
Frond development. **(A)** *L. aequinoctialis* growing in liquid culture. **(B)** Close-up view of a single frond. The locations of two budding pouches are identified by brackets: (1) the larger daughter frond and (2) the smaller daughter frond is covered by the mother frond and is out of view. **(C)** The abaxial side of a frond whose larger daughter frond (1) was removed for observation. The position of the missing frond is depicted as a dotted line. The smaller daughter frond is labeled as 2. **(D)** Fronds after germination. **(E)** Scanning electron micrograph of a budding pouch (bp). **(F)** Close-up view of **(E)** showing the early development of a third daughter frond (3). Scale bars: 1 cm **(A)**, 1mm **(B)**, 0.5 mm **(C–E)**, and 100 μm **(F)**.

To characterize vegetative organ differentiation in *L. aequinoctialis*, we first observed the development and proliferation of leaf-like structures called fronds (Figure 1A). Mature mother fronds are 3–4 mm in diameter and form two pocket-like structures called budding pouches in the proximal region (Figure 1B). Daughter fronds develop alternately in the two budding pouches, suggesting the localization of organ differentiation activity inside both budding pouches (Figure 1C). When mature, the older daughter frond detaches from the budding pouch of the mother frond, leaving a trace of the abscission zone (Figures 1D–F). In the axil of the detached zone, the next daughter frond develops (Figures 1E,F). These observations suggest that meristematic activities reside in specific regions of the budding pouch with some similarity to the axillary meristems of other angiosperms.

### *Lemna aequinoctialis* Flower Development

**Figure 2:**
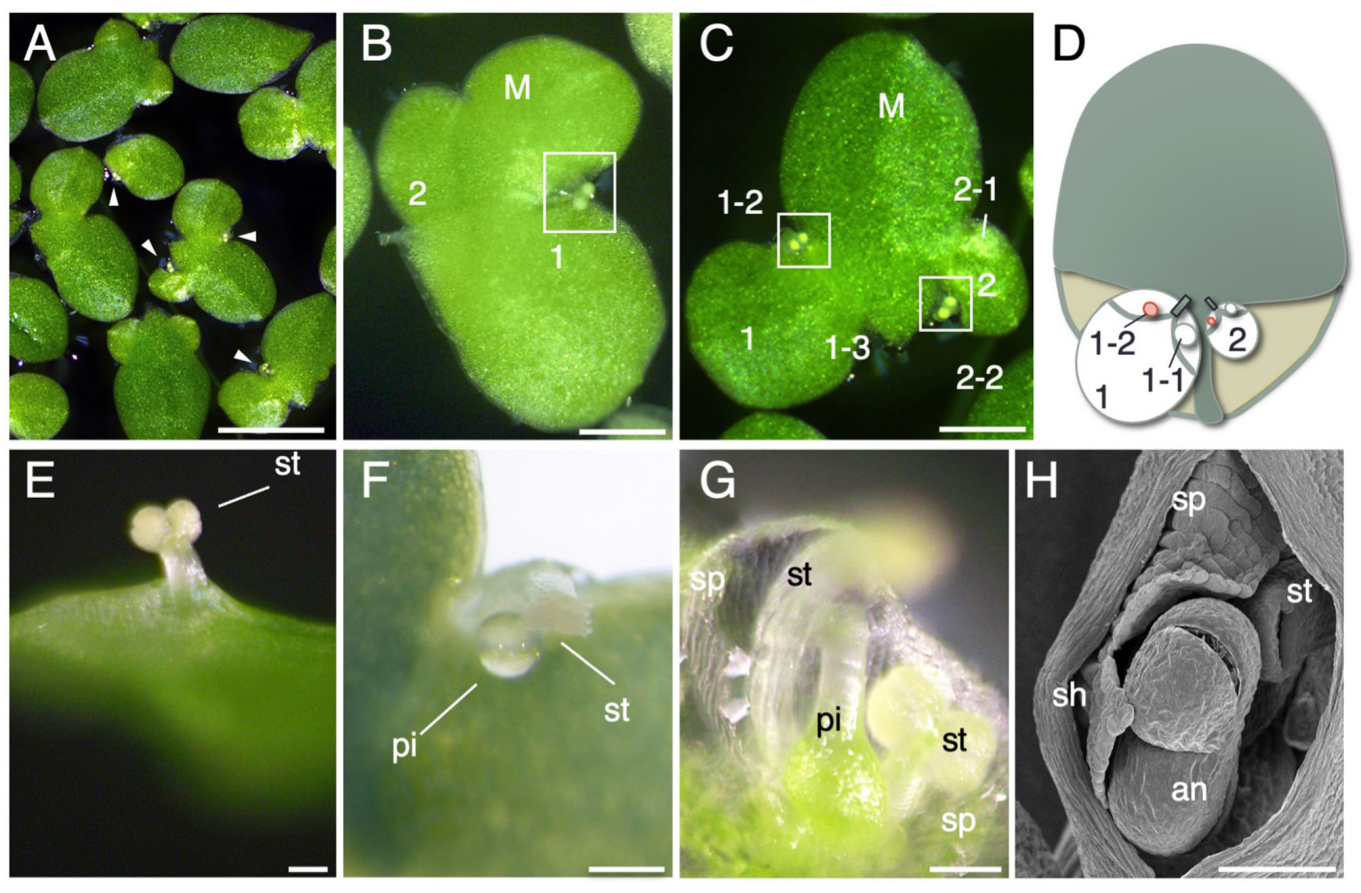
Flower development. **(A)** Flowering *L. aequinoctialis* in culture. Arrowheads indicate flowers. **(B)** Close-up view of a flowering frond. Two flowers are visible. The mother frond (M) produced two daughter fronds: a larger frond (1) and a smaller frond (2). The white rectangle indicates a flower of the larger frond (1). **(C)** Flower formation in daughter fronds. The mother frond (M) produced two daughter fronds: a larger frond (1) and a smaller frond (2). Daughter frond 1 has two budding pouches, one of which produced a granddaughter frond (1–3), and the other budding pouch produced a flower (1–2 with white rectangle). The first granddaughter frond, named 1–1, has already detached, and the picture shows 1–3, the frond that this pouch formed after 1–1. Daughter frond 2 also produced a granddaughter frond (2–1) and a flower (2–2 enclosed with a white rectangle). **(D)** Schematic diagram of **(C)**. **(E–G)** Close-up view of floral organs. an: anther, st: stamen, pi: pistil, sp: spathe. **(H)** Scanning electron micrograph of a flower. Scale bars: 1 cm **(A)**, 1mm **(B)**, and 100 μm **(C–H)**.

To characterize the pattern of flower development in *L. aequinoctialis*, we induced flowering by controlling the day length of the culture. Flowering of *L. aequinoctialis* is induced by short days (Yukawa and Takimoto, 1976). After 10 days of short-day treatment, *L. aequinoctialis* synchronously flowered (Figure 2A). Flowers of *L. aequinoctialis* are formed in the budding pouches; however, flowers are formed in only one of the two budding pouches, and a daughter frond is formed within the other budding pouch (Figure 2B). When a mother frond remains attached to two daughter fronds, each of the daughter fronds can produce a flower within one of their budding pouches, thereby producing a granddaughter frond (Figures 2C,D). Our analysis of flower development in *L. aequinoctialis* identified two distinct characteristics: the length of the stamens and the timing of stamen maturation. Most *Lemnoideae* plants develop two stamens per flower. *L. aequinoctialis* developed two stamens as shown in Figure 2G, although we found that in our culture conditions, one stamen grows rapidly and becomes longer than the other in all flowers (Figures 2E–H). Eventually, the longer stamen emerged outside the frond epidermis, whereas the smaller stamen often failed to emerge in our growth conditions (Figure 2E). The timing of stamen and pistil maturation differs among species in the *Lemnoideae*. Stamen maturation proceeds pistil maturation in some *Lemnoideae* plants, whereas the opposite timing occurs in others (Fourounjian et al., 2021). In *L. aequinoctialis*, stamens appear first (Figure 2E), then pistils appear (Figure 2F). This finding suggests the faster growth of one of the two stamens (refer Discussion).

### Identification of Regions With High Cell Proliferation Activity in *Lemna aequinoctialis*

**Figure 3:**
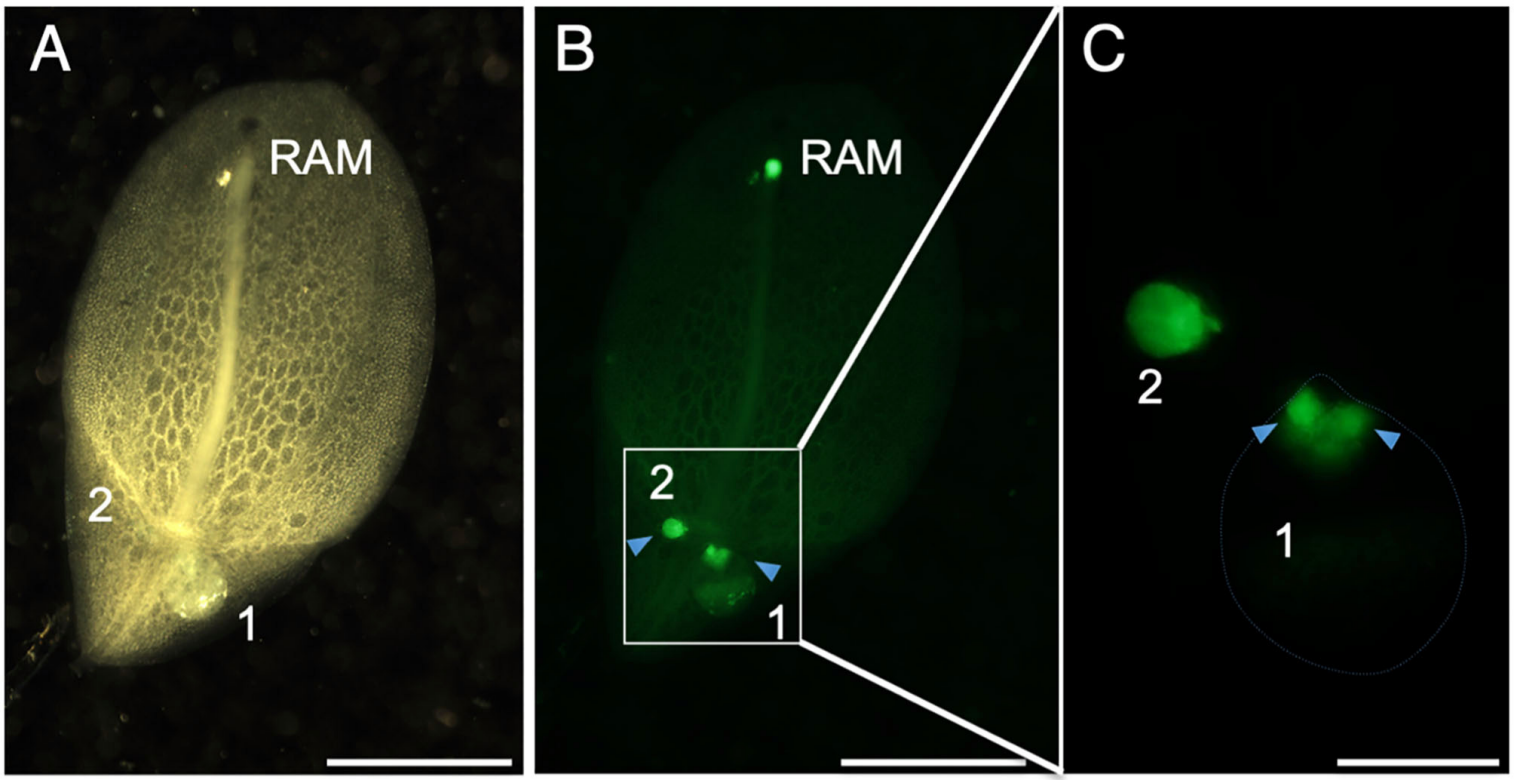
EdU. **(A)** The abaxial side of a frond stained with ethynyl deoxyuridine (EdU). **(B)** Areas of EdU fluorescence (green) are visible at the RAM and the proximal region of the frond. Arrowheads indicate separate fluorescent regions from each of the two daughter fronds (1 and 2). **(C)** A close-up view of the area enclosed by the rectangle in **(B)**. Arrowheads indicate separate fluorescent regions in daughter frond 1. Scale bars: 2 mm **(A,B)** and 0.5 mm **(C)**.

To identify regions with high cell proliferation activity, we stained *L. aequinoctialis* plants with 5-ethynyl-2'-deoxyuridine (EdU) and observed the position of the stained regions. EdU is a thymidine nucleotide analog that is incorporated into newly synthesized DNA to label cells that divided during the period of EdU application. We detected three stained regions in the root tip and proximal regions of two daughter fronds (Figures 3A,B). Staining at the root tip corresponded to the position of the root apical meristem. Close inspection of the proximal regions of the daughter fronds revealed that staining of the larger frond was divided into two separate areas (Figure 3C). These two regions were indicative of active areas of cell proliferation to generate granddaughter fronds within the budding pouches of larger daughter fronds. In contrast to the larger fronds, the small fronds stained more uniformly throughout with stronger staining in the proximal region (Figure 3C). This result suggests that active cell proliferation to form daughter fronds occurred during earlier stages. We did not detect any fluorescence indicative of the SAM in the mother frond (Figure 3C).

### *L. aequinoctialis* Transcriptome Analysis

**Figure 4:**
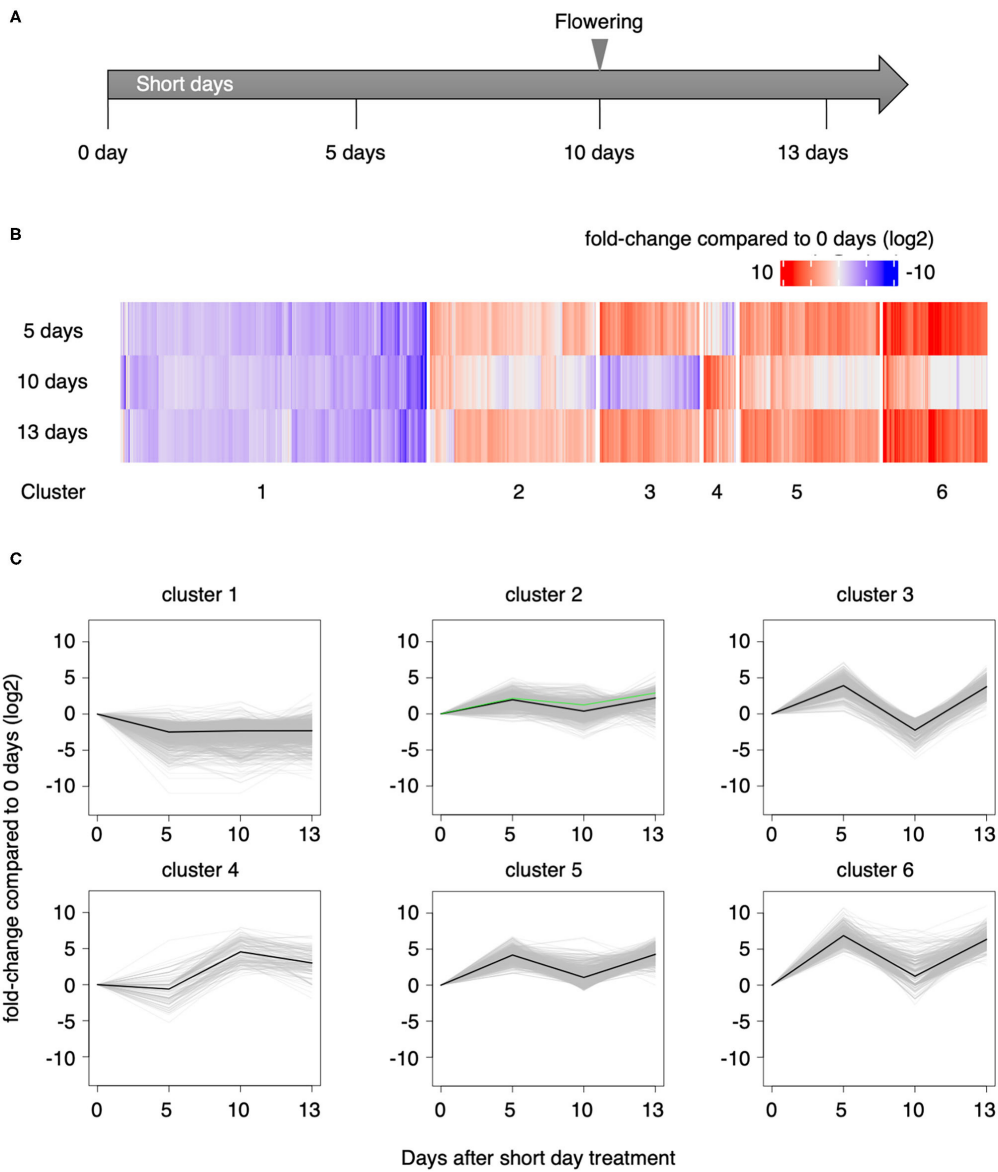
RNA-seq. **(A)** Time course of sampling *L. aequinoctialis* after initiating short-day conditions. **(B)** Heat map of gene expression changes at 5, 10, and 13 days after beginning the short-day treatment compared with the expression level on day 0. **(C)** K-means clustering of the transcriptome. The green lines in cluster 2 indicate *LaFTL1*.

To investigate gene expression patterns during the vegetative to reproductive phase change, we performed RNA-seq using triplicate samples collected on day 0 and 5 days, 10 days, and 13 days (SD0, SD5, SD10, and SD13, respectively, Figure 4A) after initiating the short-day treatment. A total of 406 million paired-end reads were sequenced, and after quality filtering, 388 million reads remained that were used for downstream analyses (Supplementary Table 1). Since the genomic sequence of *L. aequinoctialis* was not available, we conducted *de novo* transcriptome assembly with Trinity (Grabherr et al., 2011). As a result, 180,831 genes including 328,516 transcripts (N50 = 1,526 bp) were identified (Supplementary Table 2). Then, we aligned the reads against the assembled transcripts to estimate the expression levels. Between 27 and 39% of the reads were aligned to at least one locus. The average fragment we used was TransDecoder (https://github.com/TransDecoder/TransDecoder/wiki) to identify candidate protein-coding genes from all transcripts. For 35,680 genes, at least one isoform of the gene had either a partial or complete ORF with deduced proteins having lengths >100 amino acids. For functional annotation of the candidate protein-coding genes, we searched for sequence similarity against the Swiss-Prot database with BLASTp (Altschul et al., 1990). We also searched for conserved domains against the Pfam-A.hmm database with HMMER. Based on these results, 17,610 genes of 35,680 genes (49%) were annotated by TransDecoder. These genes were also annotated with GO terms.

We identified 3,908 DEGs [false discovery rate (FDR) < 0.05] during the short-day treatment (Supplementary Table 3). To dissect the expression patterns, we used K-means clustering to group the DEGs into six clusters (Figures 4B,C). Genes in clusters 2 (*N* = 1,532), 3 (*N* = 992), 5 (*N* = 1,290), and 6 (*N* = 958) behaved similarly; expression was upregulated at 5 days of short-day treatment, downregulated at 10 days, and re-upregulated at 13 days. Gene expression in cluster 4 (*N* = 290) was upregulated at 10 days of short-day treatment and was maintained or slightly reduced at 13 days. On the other hand, the expression of genes in cluster 1 (*N* = 2,824) was downregulated at 5 days and was maintained at approximately the same expression level until 13 days.

We also conducted GO term enrichment analysis for each cluster (Supplementary Table 4). In cluster 4, GO terms associated with cell wall synthesis, such as “pectin catabolic process” and “pectin metabolic process” were highly enriched. Duckweed flowered 10 days after initiating the short-day treatments. Consistent with this morphological change, many GO terms associated with “developmental process involved in reproduction” and “flower development” were also enriched.

In cluster 2 and cluster 3, many GO terms associated with “response to stimuli,” including both endogenous and environmental stimuli, were enriched. In cluster 4, several GO terms associated with “fruit ripening” were enriched. In cluster 1, several GO terms related to “photorespiration” and “photosynthesis” were enriched, suggesting an effect of the short-day treatment.

We found three *FT-like* (*FTL*) genes, TRINITY_DN5941_c0_g1 (*LaFTL1*), TRINITY_DN19284_c0_g2 (*LaFTL2*), and TRINITY_DN19135_c0_g1 (*LaFTL3*). *LaFTL1* was defined as differentially expressed and was grouped in cluster 2. This gene was upregulated at 5 days after the initiation of the short-day treatment, as shown by the green lines in Figure 4C. *LaFTL2* and *LaFTL3* also showed a similar expression pattern, although not significant.

TFL1 is a suppressor of flowering that competes with FT proteins to form transcriptional complexes with 14-3-3 (Kaneko-Suzuki et al., 2018). We identified a *TFL1* ortholog TRINITY_DN11517_c0_g1 from our transcriptome dataset and named it *LaTFL1* (Supplementary Figure 1A). LaFTL1 was defined as differentially expressed. The pattern of *LaTFL1* expression increased after 5 days of short day (SD) treatment, suggesting that the suppressive function of *LaTFL1* increases upon floral induction (refer discussion).

### Cloning of FT- and FD-Like Genes

**Figure 5:**
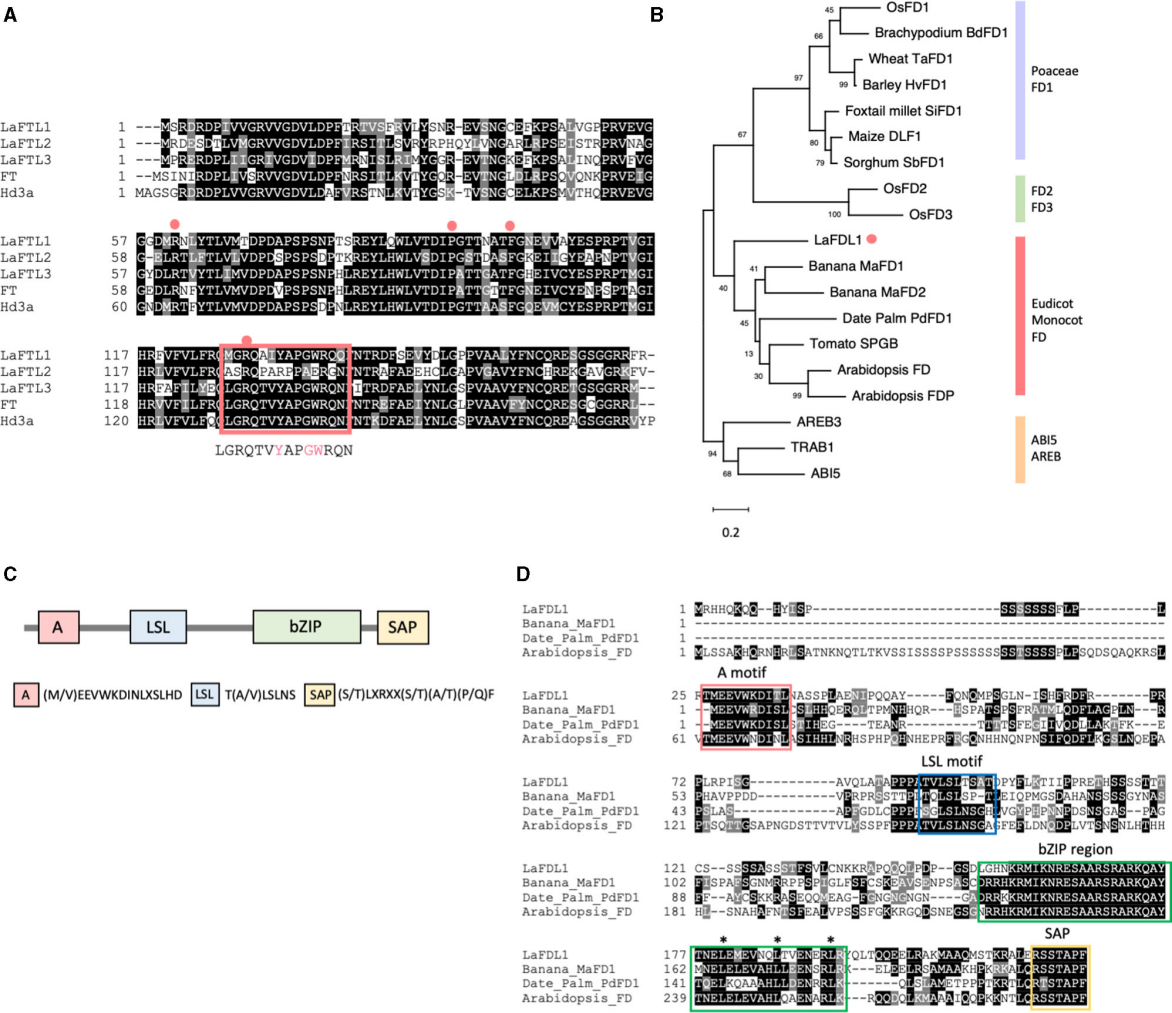
FT and FD-like proteins in L. aequinoctialis. **(A)** Deduced amino acid sequences and alignment of two FT-like proteins from *L. aequinoctialis*, rice Hd3a, and *Arabidopsis* FT. Gene IDs and accession numbers are provided in Supplementary Table 5. The four amino acid residues important for 14-3-3 protein binding in Hd3a are identified by red circles above the alignment. The red rectangle indicates the loop region of segment B. The consensus sequence for this region is shown below the alignment. **(B)** A phylogenetic tree of the FD-like protein family. The sequences were aligned by MUSCLE and the conserved residues were trimmed by trimAl. A phylogenetic analysis was conducted using the MEGA X program with 500 bootstrap replications. A maximum-likelihood tree was constructed using the Jones-Taylor-Thornton model. Representative FD-like proteins from monocots and dicots **(**Supplementary Table 5**)** were used to draw the tree. LaFDL is marked with an orange dot. **(C)** A schematic drawing of FD1 from non-Poaceae monocots FD1. The four conserved motifs (A, LSL, bZIP, and SAP) are shown. **(D)** Alignment of LaFDL1, *Arabidopsis* AtFD, Banana MaFDL1, and Date palm PpFDL1. The four conserved motifs are indicated by colored boxes. Asterisks indicate heptad repeats of leucine residues comprising the leucine-zipper motif.

In the deduced amino acid sequences of LaFTL1, LaFTL2, and LaFTL3 proteins, four amino acid residues critical for 14-3-3 protein binding (R64, P96, F103, and R132 in Hd3a) were completely conserved (Figure 5A). The segment B loop region important for floral promotion (Ahn et al., 2006; Pin et al., 2010) was also well conserved in LaFTL1 and LaFTL3; however, the segment B loop region sequence of LaFTL2 diverged from the consensus sequence. Notably, three amino acid residues essential for floral promotion (Y136, G139, and W140 in Hd3a) were substituted in LaFTL2 with R, A, and E, respectively (Figure 5A). We decided to focus on LaFTL1 and LaFTL2 in our functional analysis of LaFTLs: LaFTL1 was detected as a DEG in our transcriptome analysis, suggesting that it may be a promoter of photoperiodic flowering in *L. aequinoctialis*, while LaFTL2, although not a DEG, has interesting features in its putative amino acid sequence. The function of LaFTL3 will be analyzed in the future work.

We also identified a putative FD ortholog (LaFDL) in duckweed. A putative gene-coding region with close sequence similarity to AtFD was found in the duckweed genomic sequence, and oligonucleotides were designed to amplify the coding region from cDNA. Sequencing of the PCR products identified closely related FD-like transcripts that were designated LaFDL1 (Figure 5D). This sequence is predicted to encode a bZIP protein containing 226 amino acids. The plant FD family is divided into four subfamilies: Poaceae FD1, Eudicots and non-Poaceae monocots FD1, Poaceae FD2, and Poaceae FD3 (Figure 5B, Tsuji et al., 2013). Members of the Eudicot and non-Poaceae monocots FD1 subfamily share two conserved motifs, the A-motif and the LSL-motif, in addition to the bZIP and SAP motifs (Figure 5C). As a member of the Araceae family of monocots, Lemna's taxonomy is consistent with the classification of LaFDL1 into the Eudicots and non-Poaceae monocots FD1 subfamily (Figure 5B).

### Interaction of LaFTL and LaFDL in Yeast

**Figure 6:**
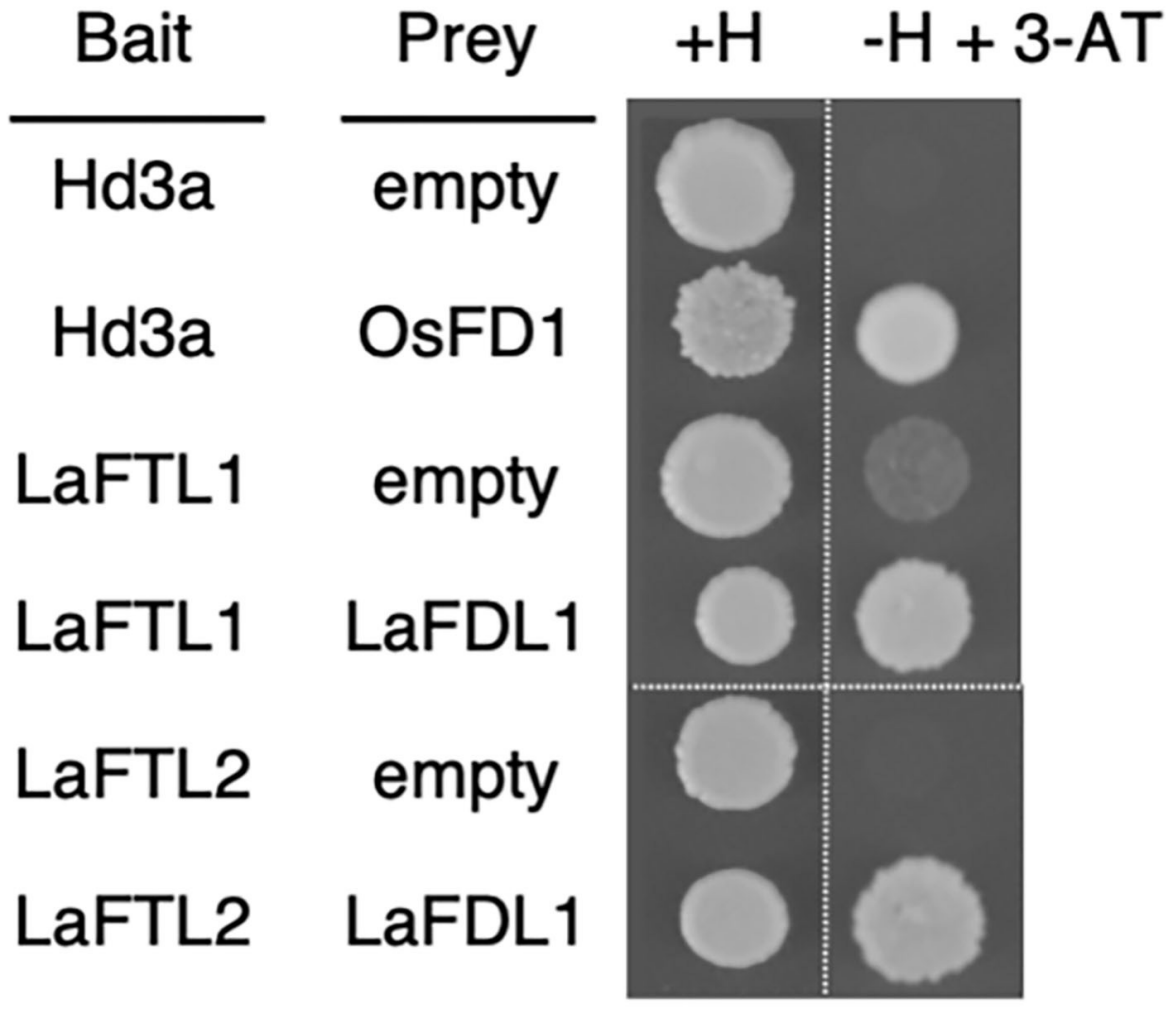
Y2H. Yeast-two hybrid results showing interactions between LaFTL and LaFDL. Yeast growth on synthetic complete medium without uracil, tryptophan, and leucine (SC-UWL) agar media containing histidine (+H) or lacking histidine plus 10 mM 3-amino-1,2,4-triazole (-H + 3-AT) is shown. Rice Hd3a-OsFD1 was included as a control.

The presence of 14-3-3 interaction motifs in LaFTL and LaFDL suggested that the deduced proteins could interact with each other through mediation by 14-3-3 proteins, similar to the formation of rice FAC. To test this possibility, we performed a yeast two-hybrid assay (Figure 6). LaFDL1 interacted with LaFTL1 and LaFTL2 with apparently similar strength (Figure 6), possibly through mediation with yeast 14-3-3 protein. This result suggested that LaFTL1 and LaFTL2 can form FAC-like complexes with LaFDL1.

### Floral Promotion Activity of LaFTL

**Figure 7:**
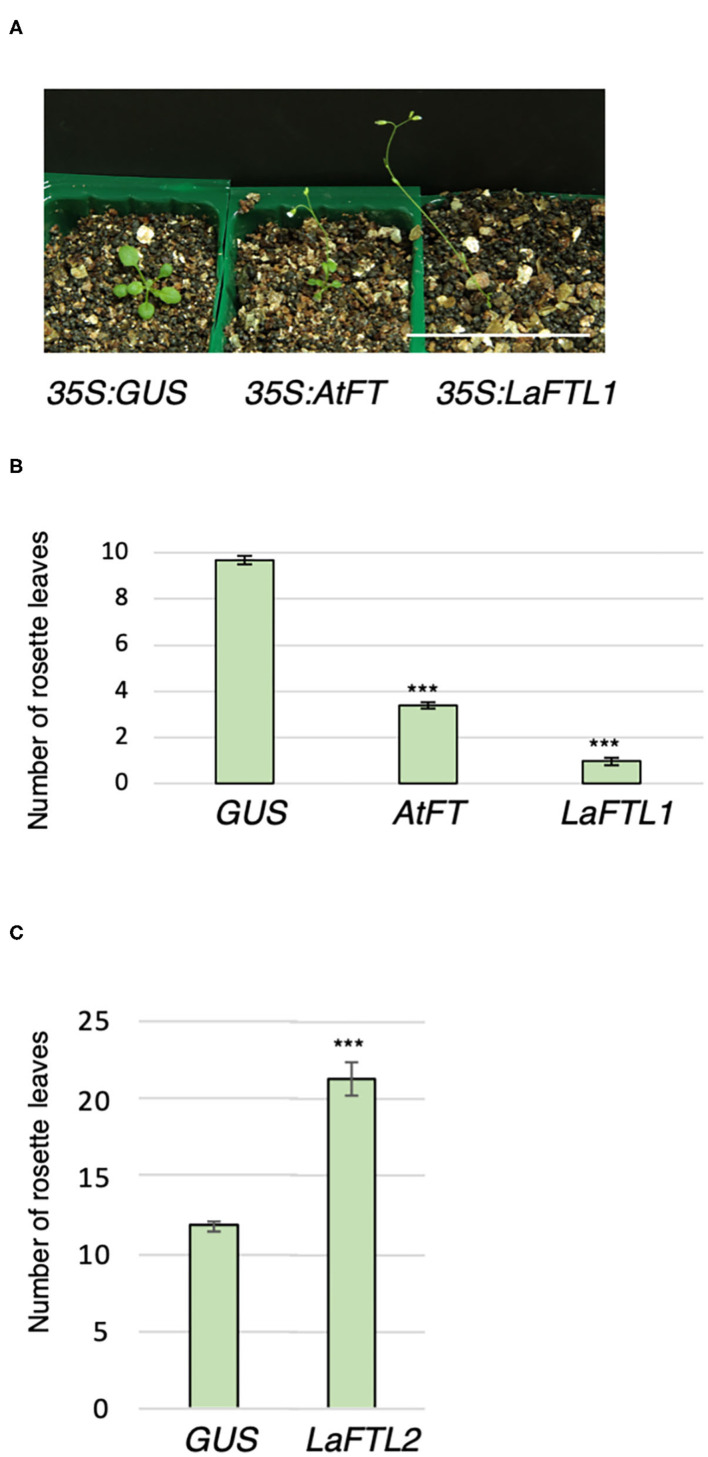
18 days LaFTL-oe in Arabidopsis. Ectopic expression of LaFTL1 accelerated flowering in *Arabidopsis*. **(A)** The flowering phenotype of transgenic *Arabidopsis* plants at 15 days after seeding. Genotypes are indicated below the photo. GUS and *Arabidopsis* AtFT were analyzed as a negative and positive control, respectively. Bar = 5 cm. **(B)** The number of rosette leaves on transgenic plants overexpressing *LaFTL1, AtFT, or GUS*. **(C)** The number of rosette leaves on transgenic plants overexpressing *GUS* or *LaFTL2*. Asterisks denote statistically significant differences determined by Student's *t*-test (*P* < 0.001). Bars indicate SE.

To assess the floral promotion activity of *LaFTL in planta, LaFTLs* were ectopically expressed under the control of the *CaMV 35S* promoter in *Arabidopsis*, and the flowering time of these transgenic plants was examined. In a previous report, *wild-type* (WT) and *35S:AtFT* plants flowered under LD conditions with 10–11 and 2–5 rosette leaves on average, respectively (Kobayashi et al., 1999). Consistently, *35S:AtFT* plants flowered with four rosette leaves on average, and 35S:GUS plants, as alternatives to WT control plants, flowered with 10 leaves on average (Figures 7A,B). *35S:LaFTL1* plants flowered earlier than *35S:GUS* plants and *35S:AtFT* plants with one-two rosette leaves. By contrast, *35S:LaFTL2* plants delayed flowering even when they had more than 20 leaves (Figure 7C). These results suggest that *LaFTL1* and *LaFTL2* function as a floral promoter and repressor, respectively.

## Discussions

In this study, we examined the morphological changes that occur during the development of fronds and flowers in *L. aequinoctialis*. We also analyzed the transcriptome of *L. aequinoctialis* after the induction of flowering, characterized the gene structure of *LaFTL* and *LaFDL*, and analyzed the function of *LaFTL*. Based on these results, we ascertained key attributes of shoot and flower development and identified the functional differentiation of FT in *L. aequinoctialis*.

### *L. aequinoctialis* Frond and Floral Development

In angiosperms, the SAM produces leaf primordia that develop into mature leaves, and axillary meristems develop within the axils of these growing leaves (Hirano and Tanaka, 2020). Fronds are considered to be part of the modified shoots of typical angiosperms. Notably, we did not find any EdU-stained fluorescence, indicating a meristematic region in the basal area of the frond where SAMs are likely to be present (Figure 3). The SAM of *L. aequinoctialis* could be aborted at an early stage of its development. *L. aequinoctialis* forms about five fronds under short-day conditions, the inducing conditions for flowering, and 14 to 20 fronds under long-day conditions, the non-inducing conditions for flowering (Landolt, 1986). These results suggest that the timing of SAM abortion is after the indicated number of frond primordia have formed. In contrast, we detected a single site of EdU fluorescence at the base of a frond with uniform staining throughout in newly formed, young, small fronds. This finding suggests that the SAM may still be maintained at the younger stage (Figure 3). Since the stipe (vascular bundle connecting the fronds) is located between the two pockets, the central part of the frond is structurally incompatible with developing the SAM. It is possible that the cells at the stronger EdU signal in the younger frond (the arrowhead on the left side of Figure 3) form the SAM (or its equivalent meristematic region).

Dichogamy is a trait in which the maturation of male and female reproductive organs is separated temporally during development (Lloyd and Webb, 1986). Protandry is a form of dichogamy in which maturation of the male state precedes that of the female state. Protandry is thought to reduce self-pollination, thereby contributing to the genetic diversity of a species (Lloyd and Webb, 1986). Examples of protandry and protogyny (the female phase proceeds the male phase) occur in Lemnoideae plants (Fourounjian et al., 2021). Our observations suggested that *L. aequinoctialis* develops two stamens, one of which grows longer; the longer stamen becomes exposed outside the frond, but, in our culture conditions, the shorter stamen often was not displayed (Figures 2E–H). The female reproductive organs grow more slowly than the stamens. In the early stages of growth, the pistil is located inside the pocket and are covered by the faster growing stamens (Figure 2H). Later, the pistils increase in size and emerge from the pocket (Figures 2F,G). That is, the order of emergence from the pockets is first the stamens, then the pistils. This finding suggests the possibility of protandry in *L. aequinoctialis*. Considering that protogyny is widely observed among Lemnoideae plants, further study will be focused on a detailed analysis of stamen and pistil maturation in *L. aequinoctialis*.

### Transcriptome Dynamics of *L. aequinoctialis* After Short-Day Treatment

We found that transcriptome dynamics after short day treatments can be summarized as an initial increase in expression levels, then a decline, followed by another increase (Figures 4B,C, clusters 2, 3, 5, and 6). The initial increase may correspond to induction of the gene expression network that promotes flowering. The decrease in expression may correspond to the suppression of flowering-related gene expression and probable initiation of flower-forming genes (Supplementary Figure 1A). This scenario is reminiscent of the expression network in Arabidopsis, where genes associated with flower development repress photoperiodic flowering genes (Liu et al., 2007). The final resurgence of expression levels of transcriptome may reflect a secondary induction of floral transition in meristematic tissue inside daughter or granddaughter fronds. This triphasic expression pattern was common to the group containing the flowering-promoting *FT* gene. Interestingly, flowering-repressing *TFL1* gene was induced by 5SD and maintained in SD10 and SD13 (Supplementary Figure 1A). This finding suggests that the flowering-promoting and the flowering-repressing gene expression networks are proceeding simultaneously during the same sampling period. After inducing flowering by SD, functional differentiation between flower-producing and frond-producing pockets was observed in the same plant, possibly reflecting this functional differentiation. Photoperiodic induction of flowering is regulated by the interaction between the circadian clock and the light signaling pathway, summarized as an external coincidence. We observed in our transcriptome that the timed expression of circadian clock genes and photoperiodic flowering-related genes overlapped. This information will be useful in further analyses to achieve a deeper understanding of the mechanism of photoperiodic flowering.

Our transcriptome analysis provided insights into the gene networks operating during photoperiodic flowering and floral development in *L. aequinoctialis*. To gain further insight into DEGs, we extracted DEGs associated with GO terms related to floral development and flowering and show their gene expression patterns as a heat map (Supplementary Figure 1B). The DEGs associated with flowering and flower development were distributed in clusters 1, 2, and 4. *LaAG* and *LaDEF/GLO* belonged to cluster 4, whose expression was downregulated at 5SD and strongly induced at 10SD and 13SD. This result suggests that the floral development program is activated between 5SD and 10SD. On the other hand, homologs of chromatin remodeling factor *SYD* (Wagner and Meyerowitz, 2002), which functions with *LFY*, were enriched in cluster 1, which contain many downregulated genes. This finding suggests that epigenetic regulators may be repressed in duckweed after induction of flowering. In addition, the expression of the transcriptional adaptor *SEUSS*, which forms a complex with *LEUNIG* and functions to repress *AG* transcription (Franks et al., 2002), was repressed after 5SD, suggesting that *SEUSS* may be involved in the induction of *AG* expression. We further investigated genes known to play important roles in these pathways in Arabidopsis, including the photoperiodic pathway genes *GI* and *CO*, FAC downstream genes *AP1, SOC1*, and *LFY*, the floral development-related genes *DEF* (*AP3*), *GLO* (*PI*), *AG*, and anti-florigen *TFL1* (Kramer et al., 1998; Abe et al., 2005; Wigge et al., 2005; Taoka et al., 2011; Kaneko-Suzuki et al., 2018). Among the identified genes, *LaAG, LaDEF/GLO*, and *LaTFL1* were included in the DEGs we identified from the time-course experiment after short-day treatment (Supplementary Figure 1). *LaAG, LaDEF/GLO* induced after 10SD, reflecting the initiation of floral organ development at this timing.

Comparing the transcriptome response to different flowering-inducing stimuli reveals integrators and other stimulus-specific networks in duckweed flowering. Fu et al. (2020) analyzed the flowering response induced by salicylic acid (SA) based on transcriptome analysis of *L. gibba*. Three genes, *LgTEM1, LgSVP*, and *LgFT1*, may play important roles in the SA-induced flowering response. Interestingly, *TEM1* and *SVP* orthologs were not detected as DEGs in our study. In contrast, *LaFTL1* was detected as a DEG in our transcriptome analysis of the short-day-induced flowering of *L. aequinoctialis*. Since *FT* was induced by two different flowering inducers (SA or daylength) in different species of the Araceae:Lemnoideae, *FT* may function as an integrator of flowering in duckweed and other plants. *TEM1* and *SVP* may constitute a gene expression network to regulate stress-responsive flowering as represented by SA treatment. The expression network for photoperiodic flowering is realized by transcriptional and posttranscriptional regulation in response to *CO*. *CO and CO-like (COL)* genes were not identified as DEGs in our analysis because the expression of *COs* is regulated in a diurnal manner, and modifier proteins regulate the accumulation or function of CO proteins. Thus, diurnal gene expression analysis or regulation of gene expression at the protein level will be important to investigate in the future.

### Diversification of FT Function in *L. aequinoctialis*

We found two orthologs of FT in *L. aequinoctialis* (Figure 5). When overexpressed in *Arabidopsis*, LaFTL1 promoted, but LaFTL2 suppressed flowering (Figure 7). The suppressive mechanism for LaFTL2 remains unclear, but the divergence in consensus sequences within the loop region of segment B could be a candidate for this activity. Divergence in segment B is known to transform the function of FT from a promoter to a suppressor of flowering in several plants including *Arabidopsis*, tomato, potato, and sugarbeet (Ahn et al., 2006; Pin et al., 2010; Abelenda et al., 2016; Soyk et al., 2017).

*L. aequinoctialis* develops two budding pouches, only one of which develops a flower during an inductive photoperiod (Figure 1). This observation suggests the presence of a limiting mechanism for flower formation in the axillary meristems of mother and daughter fronds. In the mother fronds, the SAM has aborted. In daughter fronds, one of the axillary meristems in a budding pouch is converted to a floral meristem by the activity of LaFTL1. In contrast, the SAM and the other side of the axillary meristem are protected from LaFT1 activity. Several mechanisms that protect meristems from the flower-forming activity of FT are known. The anti-florigen TFL1 is the best-known example in which TFL1 competes with FT to form protein complexes with the transcription factor FD (Kaneko-Suzuki et al., 2018; Zhu et al., 2020). A similar competitive mechanism can be applied to LaFTL2 in *L. aequinoctialis*. We showed that LaFTL2 can interact with LaFDL proteins and that LaFTL2 suppressed *Arabidopsis* flowering when overexpressed. During flowering induction, the expression of *LaFTL2* did not significantly change (Supplementary Figure 1), whereas that of *LaTFL1* did change (Figure 4). This result suggests that the presence of two types of repressors in *L. aequinoctialis*: *LaFTL2* as a constitutive repressor and *LaTFL1* as an inductive repressor. The cooperativity of *LaFTL2* and *LaTFL1* may contribute to flowering inhibition under flowering induction conditions, possibly limited to young fronds. Further study is needed to reveal whether protection from FT operates in the axillary meristems of *L. aequinoctialis*.

## Data Availability Statement

The datasets presented in this study can be found in online repositories. The names of the repository/repositories and accession number(s) can be found below: https://www.ddbj.nig.ac.jp/, DRA011840.

## Author Contributions

AY and HT conceived the research. AY conducted microscopic observations. K.Taoka conducted cloning genes, yeast two-hybrid assay, and Arabidopsis genetics. AH conducted data analysis of RNA-seq. K.Tanaka and HK conducted library preparation and sequencing of RNA-seq. TM and TO conducted isolation and maintenance of materials and provided essential information and discussion. AY and K.Toyooka conducted scanning electron micrography observation. AY, K.Taoka, AH, and HT wrote the article. All authors contributed to the article and approved the submitted version.

## Funding

This study was supported by MEXT KAKENHI, Grants-in-Aid for Scientific Research on Innovative Areas (numbers 16H06464 and 16H06466 to HT), a Grant-in-Aid for Scientific Research (A), number 16H02532, to HT, and by a Core Research for Evolutionary Science and Technology (CREST) grant from the Japan Science and Technology Agency (JST) JPMJCR16O4 to HT, and a Cooperative Research Grant of Genome Research for BioResource, NODAI Genome Research Center, Tokyo University of Agriculture.

## Conflict of Interest

The authors declare that the research was conducted in the absence of any commercial or financial relationships that could be construed as a potential conflict of interest.

## Publisher's Note

All claims expressed in this article are solely those of the authors and do not necessarily represent those of their affiliated organizations, or those of the publisher, the editors and the reviewers. Any product that may be evaluated in this article, or claim that may be made by its manufacturer, is not guaranteed or endorsed by the publisher.

## Supplementary Material

Supplementary Figure 1 **(A)** Expression patterns of *L. aequinoctialis* orthologs of photoperiodic flowering-related genes (*GI, CO, SOC1*), flower development-related genes (*AP1, DEF/GLO, AG, LFY*) and the anti-florigen gene *TFL1*. The number of days after short-day treatment is shown on the x-axis, and the logarithmically transformed count per million reads (CPM)+1 values are shown on the y-axis. **(B)** A heatmap of DEGs associated with floral development and flowering related GOs. Heatmap of log ratio of expression variation from 0SD is shown.

Supplementary Table 1 Summary of sequenced reads and alignment statistics.

Supplementary Table 2 Summary of assembled transcriptome.

Supplementary Table 3 Differentially expressed genes.

Supplementary Table 4 Gene ontology enrichment.

Supplementary Table 5 Gene IDs and Accession numbers.
