# Supplementary figures and images for: Characterization of Frond and Flower Development and Identification of FT and FD Genes From Duckweed Lemna aequinoctialis Nd
Source: Front Plant Sci. 2021 Oct 11;12:697206. doi: 10.3389/fpls.2021.697206 (PMC8542802; doi:10.3389/fpls.2021.697206)

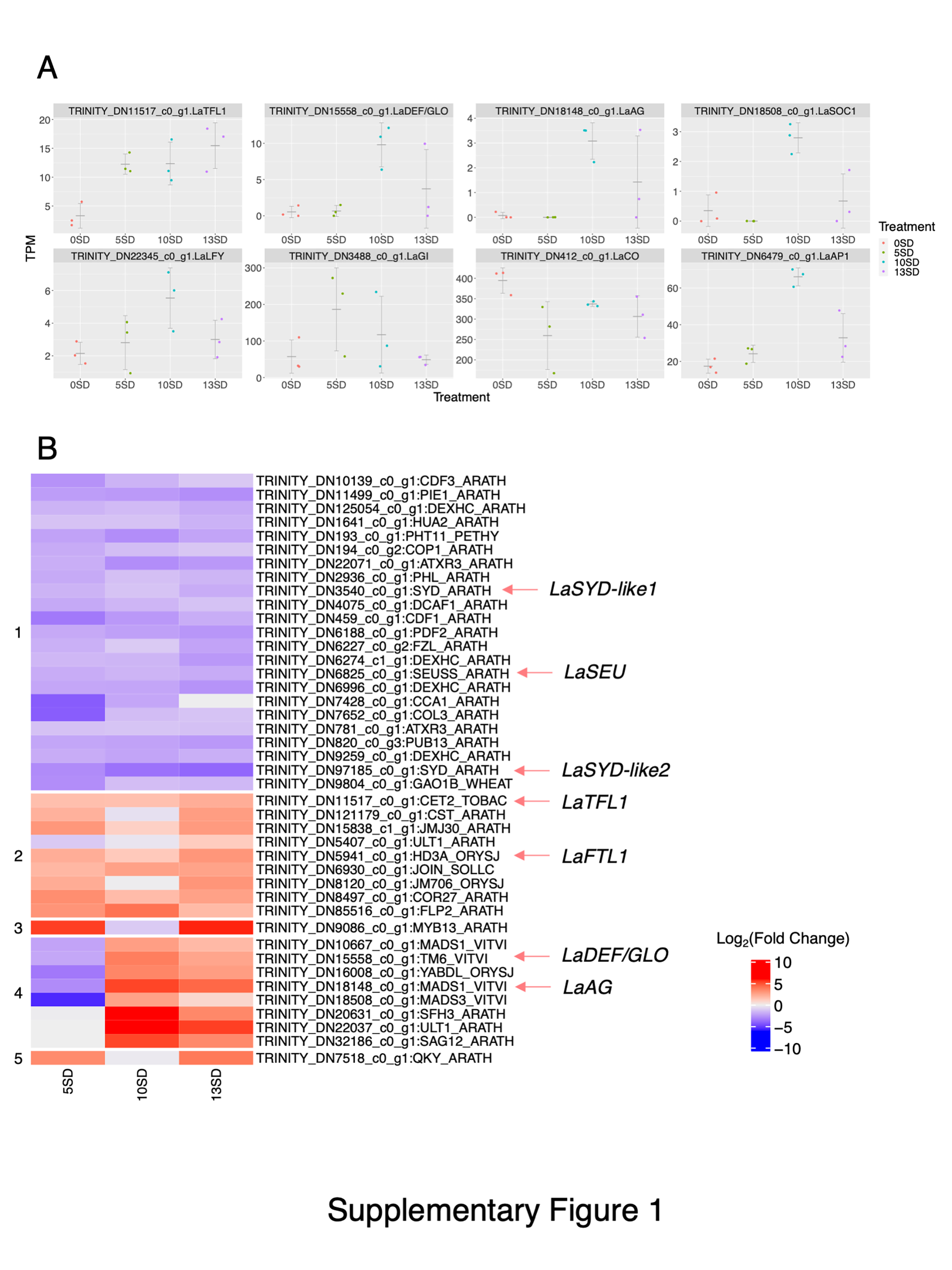

Supplement: Supplementary Figure 1 — (A) Expression patterns of L. aequinoctialis orthologs of photoperiodic flowering-related genes (GI, CO, SOC1), flower development-related genes (AP1, DEF/GLO, AG, LFY) and the anti-florigen gene TFL1. The number of days after short-day treatment is shown on the x-axis, and the logarithmically transformed count per million reads (CPM)+1 values are shown on the y-axis. (B) A heatmap of DEGs associated with floral development and flowering related GOs. Heatmap of log ratio of expression variation from 0SD is shown. [file Image_1.TIFF]
